# Supplementary material for: MetaRibo-Seq measures translation in microbiomes
Source: Nat Commun. 2020 Jun 29;11:3268. doi: 10.1038/s41467-020-17081-z (PMC7324362; doi:10.1038/s41467-020-17081-z)
Supplement: Supplementary file 10 — Supplementary Data 7 [file 41467_2020_17081_MOESM10_ESM.zip › File2/Confidence_High_Taxonomy/336792_out.krona.html]

Javascript must be enabled to view this page.

members
magnitude
magnitudeUnassigned
count
unassigned
taxon
rank

336792\_out

7

2
7
superkingdom

976
7
phylum

200643
class
7

order
7
171549

family
7
815

7
genus
816

817

SRS018313\_contig\_number\_11704SRS019397\_contig\_number\_2511SRS023346\_contig\_number\_169SRS057717\_contig\_number\_5791SRS097920\_contig\_number\_27094SRS104036\_contig\_number\_3969SRS1041039\_contig\_number\_24821
7
species
